# Associations between antidepressant therapy, work ability, and sick leave for patients with common mental disorders within a two-year perspective – A longitudinal observational cohort study in Swedish primary care

**DOI:** 10.1016/j.heliyon.2021.e07116

**Published:** 2021-05-28

**Authors:** Dominique Hange, Nashmil Ariai, Cecilia Björkelund, Irene Svenningsson, Shabnam Nejati, Eva-Lisa Petersson, Pia Augustsson, Ingmarie Skoglund

**Affiliations:** a Primary Health Care/School of Public Health and Community Medicine, Sahlgrenska Academy, University of Gothenburg, Sweden; b Research, Education, Development & Innovation, Primary Health Care, Region Västra Götaland, Sweden

**Keywords:** Antidepressant, Depression, EQ-5D, Primary care, Sick leave, WAI

## Abstract

**Background:**

An increasing number of patients are on sick leave because of common mental disorders (CMD), with or without antidepressant therapy. There is a lack of long-term follow-up studies in the primary care context, where most of the patients are treated. The importance of identifying potential factors associated with work ability for CMD patients is increasingly in focus.

**Objective:**

To investigate the associations between using antidepressants, sick leave duration, reported work ability and psychological symptoms among patients with CMD during a two-year observation period in the primary care context.

**Methods:**

Longitudinal observational cohort study at 28 Primary Care Centers in Region Västra Götaland, Sweden, including 182 patients with an employment and on sick leave for CMD. The following outcomes were assessed: work ability measured with WAI, depressive symptoms with MADRS-S, anxiety symptoms with BAI, fatigue symptoms with KEDS, quality of life with EQ-5D, and days of sick leave. The data were compared between the groups that used and did not use antidepressants, during the 24-months observation period.

**Results:**

Work ability and health-related quality of life increased over time in both groups. A steeper decrease of depressive symptoms, anxiety symptoms as well as an increased health-related quality of life at 3, 6 and 12 months was found in the group without antidepressants, although both groups levelled off at 24 months. In both groups, a higher work ability at baseline was associated with less two-year sick leave.

**Conclusion:**

Our study indicates that a high work ability at baseline has a strong association with a lower total net and gross sick leave duration during the entire two-year follow-up period for patients with CMD in primary health care, irrespective of use of antidepressants. Using WAI in primary health care could therefore be helpful in predicting return to work. Use of antidepressants during the CMD episode could indicate initially a more pronounced overall symptom pattern, motivating introduction of antidepressants, rather than prolonging the sick leave period.

## 1 Introduction

With a growing number of patients on sick leave because of common mental disorders (CMD) in most Nordic and European countries, the importance of identifying potential factors associated with sick leave is increasingly in focus [1, 2]. Concurrently, the definition of CMD has, at least in the Nordic countries, been expanded to include stress-related mental illness in addition to depression and anxiety syndromes. Several treatment options for depressive and anxiety syndromes are available in primary care, mostly antidepressant medication and less frequently psychotherapy, due to limited access in primary care. The Swedish National Board of Health and Welfare suggests cognitive behavioral therapy (CBT) as the prioritized treatment for mild to moderate states of depression as well as for anxiety syndromes in adults [3]. The availability of psychotherapy in primary care is still low and antidepressants are common [3]. Since 2006 the prescription of antidepressants has increased by 25 percent throughout the Swedish population, and in 2018 seven percent of all men and 13 percent of all women used antidepressants at some point during the year [4]. In an earlier publication from the study group “Antidepressants in Depression, Anxiety Syndromes and Stress-related Mental Disorders” (ADAS), no significant differences in sick leave duration were observed between patients with CMD treated with antidepressants compared to those treated with other therapies during a 12-month follow-up period [5].

Earlier studies have pointed out prognostic factors for return-to-work (RTW), such as patient's contact with medical specialists, RTW self-efficacy, and the individual's work ability and cognitive function, as well as environmental, social, and economic factors [6, 7]. The effectiveness of an exposure-based return-to-work (RTW-E) intervention for patients with CMD compared to care-as-usual (CAU) after 12-months follow-up showed that RTW-E had no better effects than CAU in reducing time-to-full RTW [8]. Factors that have emerged in the individual patient's perspective as important for RTW are trust in the relationship to the employer and structure and balance in the work situation [9].

Studies show that work ability does not increase to the same extent and at a similar pace as the depression lapse [10, 11, 12]. Depression is more related to poor work performance than to physical conditions [13]. Patients with CMD account for a large portion of health care, incurring considerable costs for the individual patient as well as for the society due to reduced work ability and delayed ability to RTW [14]. The largest economic detriment seems, however, to be the loss of productivity occurring after the individuals have returned to work [12, 15].

In the present study we investigated whether use of antidepressants was associated with work ability during a two-year observational period. Specific aims were to, determine whether there were differences between users and non-users of antidepressants regarding their reported work ability, and to determine whether there were associations between patients’ reported work ability and their symptoms of depression, anxiety, and stress-related mental illness, quality of life and total days of sick leave.

## 2 Materials and methods

### 2.1 Settings and subjects

From January 2014 to February 2015, a prospective cohort study, “Antidepressants in Depression, Anxiety Syndromes and Stress-related Mental Disorders” (ADAS study), was started and conducted during two years in Region Västra Götaland, Sweden [5]. All patients on sick leave for depression, anxiety, or stress-related mental illness, aged 18–60 years old, at 28 different Primary Care Centers (PCCs) were invited to participate. Patients were included if they had a minimum of 50% employment at inclusion and had been on sick leave for a duration ranging ≥14 days to 12 months. Other inclusion criteria were: diagnosed with mild to moderate depression, anxiety syndrome, or stress-related mental disorder according to the Mini International Neuropsychiatric Interview (M.I.N.I. version 6.0.0d) [16], or, concerning exhaustion disorder, diagnosed according to a review of the diagnostic criteria for the condition [17]. Exclusion criteria were persons with bipolar disorder, psychosis, schizophrenia, risk of suicide, or diagnosed with substance/alcohol dependency, ongoing severe depression, or general anxiety disorder, persons with unemployment, retirement or other reasons for not working, and persons who did not understand or speak Swedish sufficiently. All participants received verbal and written information about the study and signed an informed consent. They were also informed about the confidentiality of the data.

### 2.2 Data collection

**Figure 1:**
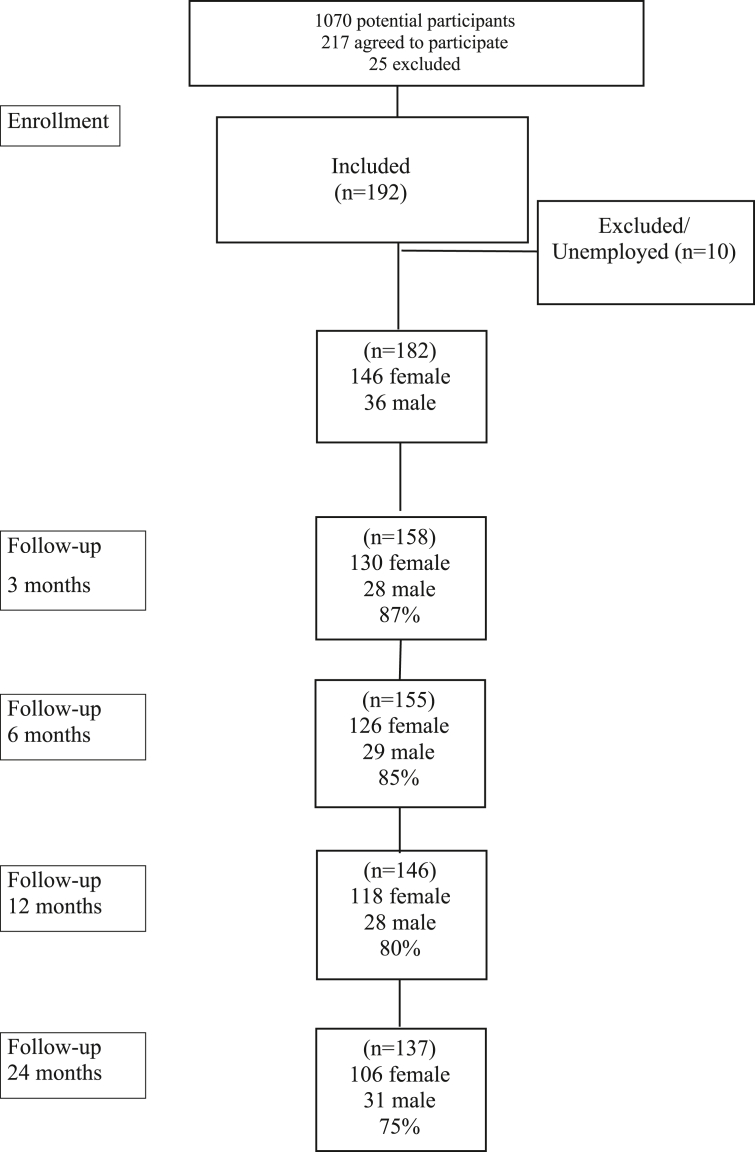
Flowchart over patient enrollment at inclusion and follow-ups, at 3, 6, 12 and 24 months.

During the inclusion period, the rehabilitation coordinator (RC) at the PCCs searched for potential participants through the IT reporting tool MedRAVE (Medrave Software AB l contact@medrave.com) about every other week. The work of an RC is primarily focused on the patients on sick leave, in coordinating the rehabilitation process and facilitate return to work. MedRAVE is a program designed for extracting data from the health care records. The first selection in MedRAVE was based on age, sick leave duration (≥14 days-12 months), and diagnosis from ICD-10, F32, F33, F34, F39, F41, F48, F49, or F99 (excluding severe depression and generalized anxiety disorder). RC asked 1070 potential participants by mail whether the patient was interested in participation and whether the research nurse could make contact. If the patient agreed to participation, information was forwarded to the research nurse, who planned for an interview at the PCCs. After oral and written information about the study, with possibilities to ask questions, the structured clinical interview with M.I.N.I. and a review of the diagnostic criteria for exhaustion disorder took place. At the clinical assessment, inclusion and exclusion criteria were considered. In all 217 patients on sick leave were recruited for the study, but 25 did not fulfill the diagnostic CMD-criteria specified for the study. Hence, a total of 192 patients were included, see flow chart Figure 1. There were no statistically significant differences between participants and non-participants concerning age, sex, marital status, SES, physical activity, sick leave or self-perceived health at baseline. Following the purpose of this study, all persons without ongoing employment (n = 10) were excluded (Figure 1).

### 2.3 Outcome measures

Patients' perceived work ability was measured by one single question (from the Work Ability Index questionnaire (WAI) (visible analogue scale) “Current work ability compared with the lifetime best”, with possible scores of 0 (“completely unable to work”) to 10 (“ability at its best”) [18, 19]. Depressive symptoms as well as the severity of the depression were measured by the Montgomery Asberg Depression Rating Scale-Self Assessment (MADRS-S) [20]. Anxiety symptoms and their intensity were measured by the Beck Anxiety Inventory (BAI) [21]. Fatigue symptoms were measured by the Karolinska Exhaustion Disorder Scale (KEDS) [22]. Health-related quality of life was measured by EuroQoL-5D (EQ-5D) [23]. All outcome measures were collected at baseline, 3, 6, 12 and 24 months. Days of sick leave, both gross and net, were collected from the patients’ questionnaires as well as from the electronic patient records and were measured during 0–3, 4–6, 7–12 and 13–24 months.

In addition, information concerning age, gender, marital status, children <18 years living at home, education, country of birth, smoking (yes/no), socioeconomic status (SES, high = senior office worker and low = lower office worker, worker, or student), and use of antidepressants (yes/no), at baseline was collected. The antidepressant use obtained by questionnaire at baseline was supplemented with data from the patients’ journals after 24 months. The patients were asked about how motivated they were to return to work during the next year, with a variable merged from two questions: “1) I am motivated to return in full or in part to my current workplace during the next year”; 2) “I am motivated to return in full or in part to work at another workplace during the next year”.

### 2.4 Instruments

The following validated and standardized scales regarding self-rated health and functioning were used at baseline: 1) Work Ability (WAI VAS): self-rated work ability - human resources in relation to health demands at work, and 2) Montgomery Asberg Depression Rating Scale-Self Assessment (MADRS-S): depressive symptoms.

In addition to the above mentioned scales for outcome measures, the following validated and standardized scales regarding self-rated health and functioning were used at baseline: 1) Beck Depression Inventory-II (BDI-II): depressive symptoms [24], 2) Hospital Anxiety and Depression Scale (HADS): degree of anxiety and depression [25], 3) Work and Social Adjustment Scale (WSAS): self-rated functional level [26], 4) Alcohol Use Disorders Identification Test (AUDIT): alcohol use [27], and 5) Satisfaction with life scale (SWLS) [28]. These instruments were used at baseline to examine whether the group who used antidepressants during the study period differed in any aspect from the group who did not use antidepressants during the study period.

### 2.5 Statistics

For continuous variables, means and standard deviations and for categorical variables, frequencies and percentages have been presented (See Table 1).

**Table 1:** Demographic characteristics at baseline for patients with and without antidepressants, as well for the total group, with numbers and percentage (%) of patients or means and standard deviation (SD).

	Total n = 182 (%)	Antidepressants n = 85 (%)	No antidepressants n = 97 (%)	p-value
**Age**				
Mean (SD)	42.9 (10.0)	43.2 (10.9)	42.6 (9.10)	0.65
**Gender**				
Male	36 (20.0)	15 (17.6)	21 (21.6)	0.50
Female	146 (80.2)	70 (82.4)	76 (78.4)	
**Marital status**				
Married or cohabiting	136 (74.7)	62 (72.9)	74 (76.3)	0.60
Single	46 (25.3)	23 (27.1)	23 (23.7)	
**Cohabiting with children <18 years**				
Yes	101 (55.5)	43 (50.6)	58 (59.8)	0.21
No	81 (44.5)	42 (49.4)	39 (40.2)	
**Education**				
Junior high school	10 (5.5)	6 (7.1)	4 (4.1)	0.48
Senior high school	92 (50.5)	45 (52.9)	47 (48.5)	
University	80 (44)	34 (40.0)	46 (47.4)	
**Born outside the Nordic countries**				
Yes	20 (11)	9 (10.7)	11 (11.3)	0.89
No	161 (89)	75 (89.3)	86 (88.7)	
**Smoking**				
Yes	40 (22)	22 (25.9)	18 (18.6)	0.23
No	142 (78)	63 (74.1)	79 (81.4)	
**SES**				
Senior office worker	77 (44.8)	30 (36.6)	47 (52.2)	0.062
Lower office worker	36 (20.9)	17 (20.7)	19 (21.1)	
Worker or student	59 (34.3)	35 (42.7)	24 (26.7)	
**Leisure-time physical activity**				
Never	27 (14.9)	14 (16.5)	13 (13.5)	
At least 4 h per week	154 (85.1)	71 (83.5)	83 (86.5)	0.58
**Sick leave at present**				
Yes	159 (87.8)	75 (89.3)	84 (86.6)	0.58
No	22 (12.2)	9 (10.7)	13 (13.4)	
**Work ability**		n = 84	n = 97	
Mean (SD)	3.2 (2.6)	3.4 (2.7)	3.1 (2.6)	0.57
**MADRS**		n = 84	n = 96	
Mean (SD)	20.6 (8.0)	20.7 (8.3)	20.5 (7.7)	0.86
**BAI**		n = 78	n = 93	0.32
Mean (SD)	20.4 (11.6)	21.4 (12.2)	19.6 (11.14)	
**KEDS**		n = 84	n = 97	0.95
Mean (SD)	27.5 (9.0)	27.4 (9.2)	27.5 (8.9)	
**EQ-5D**		n = 85	n = 97	
Mean (SD)	0.55 (0.28)	0.54 (0.30)	0.56 (0.27)	0.71
**BDI**		n = 83	n = 94	
Mean (SD)	23.1 (9.6)	23.2 (10.4)	23.1 (8.8)	0.94
**HAD anxiety**		n = 83	n = 97	
Mean (SD)	10.7 (4.1)	10.7 (4.0)	10.7 (4.1)	0.99
**HAD depression**		n = 84	n = 97	
Mean (SD)	8.6 (4.4)	8.4 (4.4)	8.7 (4.4)	0.64
**WSAS**		n = 82	n = 96	
Mean (SD)	23.2 (8.2)	23.6 (8.5)	22.8 (8.0)	0.55
**Audit**		n = 75	n = 87	
Mean (SD)	3.9 (4.2)	4.1 (5.0)	3.7 (3.4)	0.59
**SWLS**		n = 82	n = 96	
Mean (SD)	20.0 (6.3)	19.3 (6.3)	20.6 (6.3)	0.17

Continuous variables were analyzed by independent sample t-test or Mann-Whitney U test and categorical variables or frequencies by using Pearson chi-square test.

**Figure 2:**
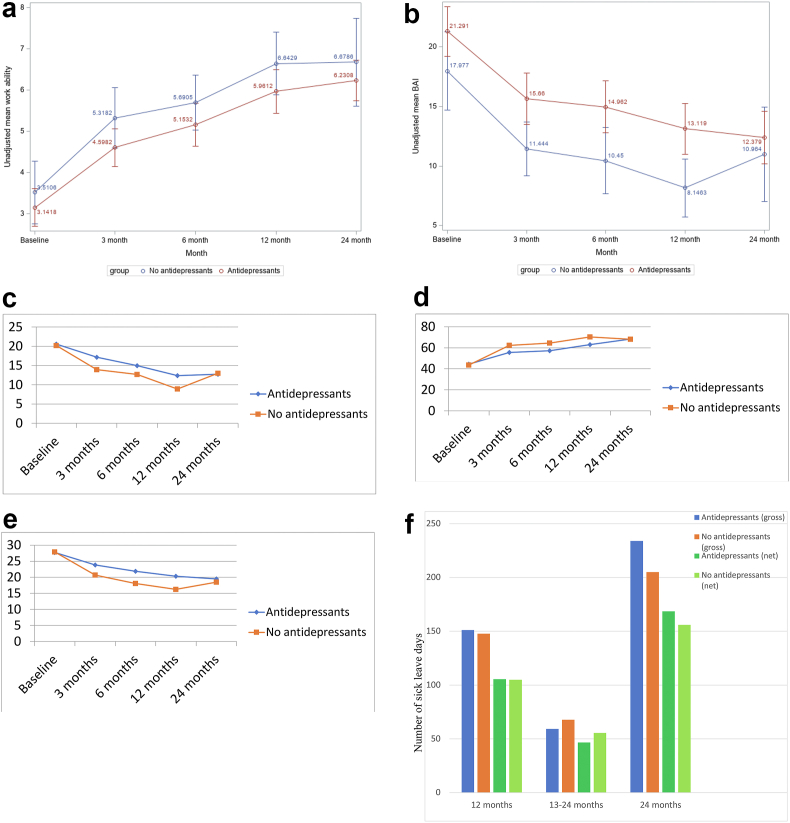
(a): Unadjusted mean values of work ability from baseline to 3, 6, 12 and 24 months in the groups with and without antidepressants use during 24 months observation period. (b): Unadjusted mean values of BAI from baseline to 3, 6, 12 and 24 months in the groups with and without antidepressants use during 24 months observation period. (c): Adjusted mean values of MADRS-S from baseline to 3 (p < 0.05), 6 (p = 0.09), 12 (p < 0.05) and 24 (p = 0.89) months in the groups with and without antidepressants. Data analyses were adjusted for age, sex, SES (high/low at baseline), antidepressants at baseline, self-perceived health at baseline, motivation to return to work during the next year, and response variable at baseline. Abbreviations: MADRS, Montgomery Asberg Depression Rating Scale. (d): Adjusted mean values of EQ-5D (1-100p) from baseline to 3 (p < 0.05), 6 (p < 0.05), 12 (p < 0.05), and 24 (p = 0.96) months in the groups with and without antidepressants. Data analyses were adjusted for age, sex, SES (high/low at baseline), antidepressants at baseline, self-perceived health at baseline, motivation to return to work during the next year, and response variable at baseline. Abbreviations: EQ-5D EuroQoL-5D, health-related quality of life. (e): Adjusted mean values of KEDS from baseline to 3 (p < 0.05), 6 (p < 0.05), 12 (p < 0.01) and 24 (p = 0.58) months in the groups with and without antidepressants. Data analyses were adjusted for age, sex, SES (high/low at baseline), antidepressants at baseline, self-perceived health at baseline, motivation to return to work during the next year, and response variable at baseline. Abbreviations: KEDS, Karolinska Exhaustion Disorder Scale. (f): Adjusted mean values of sick leave, gross/net, at 12, 13–24 and 24 months in the groups with and without antidepressants. Data analyses were adjusted for age, sex, SES (high/low at baseline), antidepressants at baseline, self-perceived health at baseline, the size of primary care center, motivation to return to work during the next year, and response variable at baseline.

Means of work ability, depressive symptoms, quality of life, KEDS, BAI and sick leave days were compared between the antidepressant users and non-antidepressant users by using independent sample t-test or Mann-Whitney U test and mixed model analysis with repeated measures for considering some variables at baseline. Data for work ability and BAI are presented in Figures 2a, b. Data for depressive symptoms, quality of life, KEDS and sick leave days in Figures 2c–f.

Means of intra-individual change of work ability, depressive symptoms, quality of life, KEDS, BAI from baseline to 3, 6, 12, and 24 months and sick leave days from 13–24 months, were compared between the antidepressant users and non-antidepressant users by using independent sample t-test or Mann-Whitney U test and GLM variance analysis for adjusting of some variables at baseline (Table 2).

**Table 2:** Unadjusted mean values of delta-WAI, delta-MADRS-S, delta-EQ-5D, delta-KEDS, delta-BAI from baseline to 3, 6, 12 and 24 months and delta-sick leave from 13 to 24 months.

Variables	Antidepressants	No antidepressants	p-value
	Mean (SD)	Mean (SD)	
**Delta-WAI**			
Baseline to 3 months	−1.22 (2.50)	−2.00 (2.67)	**0.008**
Baseline to 6 months	−1.82 (2.77)	−2.40 (2.92)	0.070
Baseline to 12 months	−2.78 (3.29)	−3.00 (3.06)	0.682
Baseline to 24 months	−3.00 (3.67)	−2.54 (3.88)	0.559
13 to 24 months	−0.40 (3.78)	0.30 (3.56)	0.359
**Delta-MADRS-S**			
Baseline to 3 months	2.70 (6.42)	5.96 (8.26)	**0.006**
Baseline to 6 months	5.14 (7.44)	7.71 (7.35)	**0.038**
Baseline to 12 months	7.67 (7.88)	11.03 (8.76)	**0.017**
Baseline to 24 months	7.63 (12.39)	7.52 (12.49)	0.967
13 to 24 months	−0.531 (12.26)	−3.45 (13.99)	0.270
**Delta-EQ-5D**			
Baseline to 3 months	−10.08 (18.46)	−18.53 (19.73)	**0.006**
Baseline to 6 months	−13.45 (21.59)	−18.83 (22.44)	0.140
Baseline to 12 months	−18.88 (25.33)	−24.60 (22.68)	0.162
Baseline to 24 months	−23.85 (29.57)	−25.21 (24.32)	0.821
13 to 24 months	−5.56 (28.44)	−0.353 (24.27)	0.352
**Delta-KEDS**			
Baseline to 3 months	3.88 (5.92)	6.49 (7.67)	**0.020**
Baseline to 6 months	5.87 (8.62)	9.05 (9.04)	**0.033**
Baseline to 12 months	7.19 (9.31)	11.03 (10.03)	**0.019**
Baseline to 24 months	7.82 (14.12)	9.38 (15.06)	0.605
13 to 24 months	1.16 (15.35)	−1.24 (17.05)	0.465
**Delta-BAI**			
Baseline to 3 months	5.29 (9.46)	6.79 (8.30)	0.316
Baseline to 6 months	5.92 (10.26)	8.28 (9.16)	0.172
Baseline to 12 months	7.25 (11.03)	9.68 (11.16)	0.207
Baseline to 24 months	8.48 (18.16)	7.23 (11.60)	0.739
13 to 24 months	0.91 (16.07)	−2.03 (13.0)	0.363
**Delta-Sick leave**			
13 to 24 months gross	88.57 (136.73)	76.29 (107.45)	0.258
13 to 24 months net	53.73 (98.52)	48.37 (73.71)	0.243

Abbreviations: MADRS, Montgomery Asberg Depression Rating Scale; EQ-5D EuroQoL-5D, health-related quality of life; KEDS, Karolinska Exhaustion Disorder Scale; BAI, Beck Anxiety Inventory.

Bold values denote p <0.05.

Linear regression analyses were used to assess relationships between work ability and MADRS-S, EQ-5D, KEDS, BAI and sick leave with baseline values and at 3, 6, 12 and 24 months with and without adjustment for some variables at baseline (Table 3). Linear regression analyses were used as well to assess relationships longitudinally between work ability at baseline and MADRS-S, EQ-5D, KEDS, BAI and sick leave days measured at 12 and 24 months with and without adjustment for some variables at baseline (Table 4).

**Table 3:** Linear regression analysis between work ability and MADRS-S, EQ-5D, KEDS, BAI and sick leave with baseline values and at 3, 6, 12 and 24 months.

Variables	WAI	95% CI	p-value
	B		
MADRS-S			
Baseline	−0.155	−0.198 to −0.113	<0.001
3 months	−0.125	−0.165 to −0.085	<0.001
6 months	−0.194	−0.228 to −0.159	<0.001
12 months	−0.218	−0.256 to −0.180	<0.001
24 months	−0.166	−0.203 to −0.128	<0.001
EQ-5D (1-100p)			
Baseline	0.079	0.063 to 0.095	<0.001
3 months	0.085	0.070 to 0.101	<0.001
6 months	0.088	0.074 to 0.101	<0.001
12 months	0.093	0.079 to 0.107	<0.001
24 months	0.099	0.085 to 0.113	<0.001
KEDS			
Baseline	−0.143	−0.181 to −0.105	<0.001
3 months	−0.146	−0.180 to −0.111	<0.001
6 months	−0.183	−0.212 to −0.155	<0.001
12 months	−0.179	−0.206 to −0.152	<0.001
24 months	−0.168	−0.195 to −0.141	<0.001
BAI			
Baseline	−0.044	−0.079 to −0.010	0.011
3 months	−0.053	−0.090 to −0.016	0.006
6 months	−0.117	−0.153 to −0.081	<0.001
12 months	−0.140	−0.176 to −0.105	<0.001
24 months	−0.132	−0.167 to −0.098	<0.001
Sick leave	**WAI with baseline**		
12 months gross	−0.008	−0.010 to −0.005	<0.001
24 months total gross	−0.004	−0.005 to −0.002	<0.001
12 months net	−0.010	−0.013 to −0.007	<0.001
24 months total net	−0.004	−0.006 to −0.002	<0.001

Abbreviations: WAI, Work Ability; MADRS, Montgomery Asberg Depression Rating Scale; EQ-5D EuroQoL-5D, health-related quality of life; KEDS, Karolinska Exhaustion Disorder Scale; BAI, Beck Anxiety Inventory.

**Table 4:** Linear regression analysis longitudinally between work ability at baseline and MADRS-S, EQ-5D, KEDS, BAI and sick leave measured at 12 and 24 months.

Variables	WAI at baseline	95% CI	p-value
	B		
MADRS-S			
12 months	−0.056	−0.109 to −0.003	**0.037**
24 months	−0.003	−0.053 to 0.047	0.909
EQ-5D (1-100p)			
12 months	0.024	0.003 to 0.045	**0.027**
24 months	0.002	−0.021 to 0.025	0.848
KEDS			
12 months	−0.047	−0.088 to −0.006	**0.025**
24 months	0.001	−0.040 to 0.042	0.968
BAI			
12 months	−0.012	−0.057 to 0.032	0.588
24 months	0.002	−0.039 to 0.044	0.909
Sick leave			
12 months gross	−0.008	−0.010 to −0.005	**< 0.001**
24 months total gross	−0.004	−0.005 to −0.002	**< 0.001**
12 months net	−0.010	−0.013 to −0.007	**< 0.001**
24 months total net	−0.004	−0.006 to −0.002	**< 0.001**

Abbreviations: WAI, Work Ability; B, beta coefficient; MADRS, Montgomery Asberg Depression Rating Scale; EQ-5D EuroQoL-5D, health-related quality of life; KEDS, Karolinska Exhaustion Disorder Scale; BAI, Beck Anxiety Inventory.

Bold values denote p <0.05.

All data analyses except descriptive analysis (Table 1) were adjusted for age, sex, SES (high/low at baseline), antidepressants at baseline, self-perceived health at baseline, motivation to return to work during the next year.

Statistical analyses were conducted using statistical software SPSS, version 25 and SAS, version 9.4. Statistical significance was set at p < 0.05.

### 2.6 Power calculation

Assumption was based on an approximate measure obtained through a register-based study [29]. With a significance level of 0.05 and power estimate of 0.80 (β = 0.20), 168 patients were required for analysis, also considering sex, socio-economic status, and environmental factors at work.

### 2.7 Ethical approval and participants’ consent

The ADAS study was approved by the Regional Ethical Review Board in Gothenburg, Sweden (Dnr 577-13, 2013-11-18). The participants received verbal and written information about the study, and the confidentiality of the data. All participants signed an informed consent form, in accordance with the provisions of the Helsinki Declaration.

## 3 Results

The study included 182 patients, all of whom stated that they were employed. Participant rate at 3, 6, 12 and 24 months was 87%, 85%, 80%, and 75% (Figure 1).

Number of patients on antidepressants at baseline was 85 (47%). The use of antidepressants varied during the long follow-up period. Regarding the first 12 observation months, 109 patients in total were prescribed and used antidepressant medication at some time during this period. About the entire observation period of 24 months, 135 patients in total used antidepressants at some time during this period. Other treatment such as psychological treatment or physical activity could be present in both groups.

Demographic data concerning, age, gender, marital status, education level, employment, and sick leave status are shown in Table 1. There were no significant differences at baseline between the groups with and without use of antidepressants during the observation period. The mean age of participants was 43 years (SD = 10.0) and 80 % were women, with slightly more men in the group without antidepressants than in the group with antidepressants. After 3 months, 66% of the patients had returned to work, corresponding to 72% after 6 months, 77% after 12 months, and 87.5% at 24 months. Return to work was defined for all patients on 100% sick leave at baseline who returned to work to some degree after 3, 6, 12 or 24 months.

Follow-up concerning work ability (WAI VAS) and anxiety symptoms at 3, 6, 12 and 24 months is presented in Figure 2a, b. WAI increased over time in both the group with antidepressants and the group without antidepressants.

Depressive, anxiety and fatigue symptoms decreased in both groups. There was a significant difference in decrease of anxiety symptoms (BAI) at 6 (p = 0.019) and 12 (p = 0.016) months between the groups, with a steeper decrease in the group without antidepressants, although this levelled off at 24 months (Figure 2b). There was also a significant difference in increase of health-related quality of life at 3, 6 and 12 months, with a steeper increase in the group without antidepressants, although this also levelled off at 24 months (Figure 2d).

All data analyses were adjusted for age, sex, self-perceived health at baseline, SES (high/low at baseline), antidepressants at baseline, and, for sick leave, motivation to return to work during the next year. However, since there were no major differences in the results of data analysis between with and without adjusting for baseline variables, we choose to show results without adjustments, i.e. crude data. Table 2 shows change of mean values between the 0–3, 4–6, 7–12 and 13–24 month follow-up of: Δ-WAI, ΔMADRS-S, ΔEQ-5D, ΔKEDS, ΔBAI, and Δ-sick leave in the groups, where both ΔMADRS-S and ΔKEDS were significantly higher at 3 and 12 months in the group without antidepressants, but this difference disappeared at 24 months.

There was an association between higher WAI and MADRS-S, KEDS, and BAI, at 3, 6, 12 and 24 months, where the beta was negative (i.e. perceived functionality increased as the depressive, anxiety and fatigue symptoms decreased) (Table 3). Concerning EQ-5D in relation to WAI, there was a significant increase during the 24 months (i.e. a positive beta value means that when the functionality increases, the health-related quality of life also increases). There was also a significant relationship between higher WAI at baseline and fewer days of sick leave during the entire follow-up period of 24 months (Tables 3 and 5).

**Table 5:** Mean values WAI (1-10 p) for return to work (Yes/No) after 3, 6, 12 and 24 months.

Variables	N (%)	WAI (1-10 p) Mean (SD)	p-value
RTW after 3 months (Yes)	59 (66)	5.0 (2.1)	<0.001
RTW after 3 months (No)	30 (34)	2.2 (1.9)	
RTW after 6 months (Yes)	61 (72)	5.5 (2.1)	<0.001
RTW after 6 months (No)	24 (28)	1.6 (1.9)	
RTW after 12 months (Yes)	61 (77)	6.5 (2.1)	<0.001
RTW after 12 months (No)	18 (23)	2.4 (2.1)	
RTW after 24 months (Yes)	56 (87.5)	7.1 (1.8)	0. 022
RTW after 24 months (No)	8 (12.5)	3.0 (3.6)	

There was an association between ΔWAI and ΔMADRS-S (p < 0.001), ΔEQ-5D (p < 0.001), and ΔKEDS (p < 0.001), for all participants during the 24 months. For ΔWAI and ΔBAI, there was an association from baseline to 6, 12, and 24 months (p < 0.001). No associations could be seen between ΔWAI and Δsick leave (gross/net). Adjustment for age, gender, SES (high/low), antidepressants, self-perceived health, motivation to return to work during the next year, and the respective response variable at baseline did not change the results to a considerable extent. The association between ΔWAI and ΔMADRS-S, ΔEQ-5D, and ΔKEDS remained significant (p < 0.001) (Data not shown).

Further, there was a significant association between WAI at baseline and sick leave, both gross and net, at 12 as well as 24 months (see Table 4). MADRS-S, EQ-5D, and KEDS levels were significantly associated to WAI at baseline, but not at 24 months (Table 4).

## 4 Discussion

The aim of this longitudinal observational cohort study was to investigate whether use of antidepressants among patients on sick leave due to CMD was associated with their reported work ability during a two-year period. We also wanted to determine whether there were associations between patients’ work ability and their depressive symptoms, stress-related mental illness, quality of life and days of sick leave. The most important finding was that most of the patients recovered from their CMD during the 24-month follow-up period, regardless of whether they had used antidepressants or not. Use of antidepressants during the CMD episode seems to indicate initially a more pronounced overall symptom pattern, motivating introduction of antidepressants, rather than prolonging the sick leave period.

The group with antidepressants showed a tendency, albeit not significant, to have a somewhat shorter sick leave period than the group without antidepressants, in contrast to Bryngelsson et al in their register-based study, who found that patients with antidepressants returned to work later than those without antidepressant [29]. It is impossible to determine whether this was because of the antidepressants, or more likely, that they did not recover as rapidly as expected from their CMD and therefore received antidepressants.

Some patients with CMD in our study were not treated with antidepressants at the beginning of their illness but received them later. Their recovery rate seems to have determined the treatment that they would receive. Patients who did not recover within an expected period or who even felt worse could receive antidepressants. This has been shown by Hyde et al, where the preferred strategy of many GPs was active expectancy, but antidepressants were prescribed earlier when symptoms were perceived to be persistent, and their decisions considered both clinical and social criteria [30].

Work ability increased over time, both in the group with antidepressants and in the group without antidepressants, which has also been seen in other patients with similar diagnoses but other types of treatment in primary care [31].

Since there are few long-term follow-up studies of patients with CMD in the primary care context, we have compared our findings with other types of studies. Nielsen et al found that employees sick-listed with self-reported stress/burnout returned to work more quickly than those with self-reported depression (antidepressant use not mentioned) after one year [32]. Our study included patients with depression, anxiety and/or stress-related mental illness, although not self-reported, and the design of the study could not detect any such differences. In a population study of CMD and long-term sickness absence with a follow-up of >6 years, although the participants were not on sick leave at baseline, CMD were long-term predictors of onset, duration, and recurrence of sick leave [33].

In our study, both groups had a significant association between higher WAI at baseline and lower gross and net sick leave during the entire two-year follow-up period. This has been described previously in register-based studies [34, 35], but it is important to confirm this result in clinical studies in primary health care. WAI could be used by GPs in the consultation with patients with CMD as a predictor of sick leave duration.

Depressive, anxiety, and fatigue symptoms decreased in both groups. There was a significant difference in decrease of depressive symptoms (MADRS-S) at 3 and 12 months, with a steeper decrease in the group without antidepressants, although this levelled off at 24 months. An untreated depression can last up to 12 months [36].

We could not show any differences in either gross or net sick leave days between the two groups during the entire follow-up period. The length of sick leave was associated with WAI at baseline. Patients with high levels of WAI at baseline returned earlier to work compared to patients with low levels of WAI, which has also been shown by Bethge at al., where persons with poor baseline work ability had 12.2 times higher odds of prolonged sick leave after 12 months [37]. An important focus for future research is to determine whether there is an instrument that can predict those patients with CMD who are at a higher risk for a long sick leave. It is essential to identify more individualized strategies that can improve health related quality of life as well as possibilities to return to work. For example, the Danish IBBIS randomized controlled trial will investigate whether integrated mental health care and vocational rehabilitation can improve return to work rates for people on sick leave because of CMD [38], and our research group has similar ongoing studies [39, 40, 41].

### 4.1 Methodological considerations

The strengths of this study are the primary care context where the majority of patients with CMD are treated, the high participation rate during the long-term follow-up for 24 months, and the fact that the patients have a well-defined diagnosis.

Among the limitations of the study are that only around 20% men participated and that patients who had language difficulties and/or serious mental health problems were not included and therefore are not represented in the material.

## 5 Conclusion

Our study indicates that a high work ability at baseline has a strong association with lower sick leave duration during the entire follow-up period of two years for patients with CMD in primary health care, with or without treatment with antidepressants. Using WAI in primary health care can be helpful in predicting RTW.

Use of antidepressants during the CMD episode seems to indicate initially a more pronounced overall symptom pattern, motivating introduction of antidepressants, rather than prolonging the sick leave period. Patients on antidepressants might have a different expression of their CMD, which is why it is important to work patient-centered in order to determine the correct diagnosis as well as treatment for each patient in order to promote recovery and return to work.

## Declarations

### Author contribution statement

Cecilia Björkelund: Conceived and designed the experiments; Analyzed and interpreted the data.

Ingmarie Skoglund: Conceived and designed the experiments; Performed the experiments; Analyzed and interpreted the data.

Dominique Hange: Conceived and designed the experiments; Performed the experiments; Analyzed and interpreted the data; Wrote the paper.

Shabnam Nejati and Pia Augustsson: Performed the experiments; Contributed reagents, materials, analysis tools or data.

Nashmil Ariai, Eva-Lisa Petersson and Irene Svenningsson: Analyzed and interpreted the data.

### Funding statement

This work was supported by Region Västra Götaland, Sweden and the Swedish Society of Medicine.

### Data availability statement

Data will be made available on request.

### Declaration of interests statement

The authors declare no conflict of interest.

### Additional information

No additional information is available for this paper.
